# Novel polyimides containing flexible carbazole blocks with electrochromic and electrofluorescencechromic properties†

**DOI:** 10.1039/c9ra10515h

**Published:** 2020-02-14

**Authors:** Rongrong Zheng, Tao Huang, Zhipeng Zhang, Zhiyao Sun, Haijun Niu, Cheng Wang, Wen Wang

**Affiliations:** Key Laboratory of Functional Inorganic Material Chemistry, Ministry of Education, Department of Macromolecular Science and Engineering, School of Chemical, Chemical Engineering and Materials, Heilongjiang University Harbin 150080 P. R. China wangc_93@163.com haijunniu@hotmail.com; School of Materials Science and Engineering, Harbin Institute of Technology Harbin 150080 P. R. China

## Abstract

A series of polyimides (PIs) were prepared by polycondensation of a diamine monomer with five anhydrides (1,2,4,5-benzenetetracarboxylic anhydride (BTA), 1,4,5,8-naphthalenetetracarboxylic dianhydride (NTD), 3,3′,4,4′-biphenyltetracarboxylic dianhydride (BTD), 4-[(1,3-dihydro-1,3-dioxo-5-isobenzofuranyl)oxy]-1,3-isobenzofurandione (DDII), and 3,3′,4,4′-benzophenonetetracarboxylic dianhydride (BPTD)), which have anodic electrochromic (EC) properties. These PIs not only show good solubility and thermal stability, but also demonstrate stable electrochemical oxidation behavior and good EC properties, and the highest retained electroactivity reaches 99% after 600 cycles. In addition, the series of PIs exhibit excellent electrofluorescencechromic (EFC) properties. Therefore, the novel materials will contribute to the application of EC or EFC displays in the future.

## Abstract 2

Polyimides prepared by polycondensation of new diamine with different anhydrides have electrochromic and electrofluorochromic properties.

## Graphical abstract

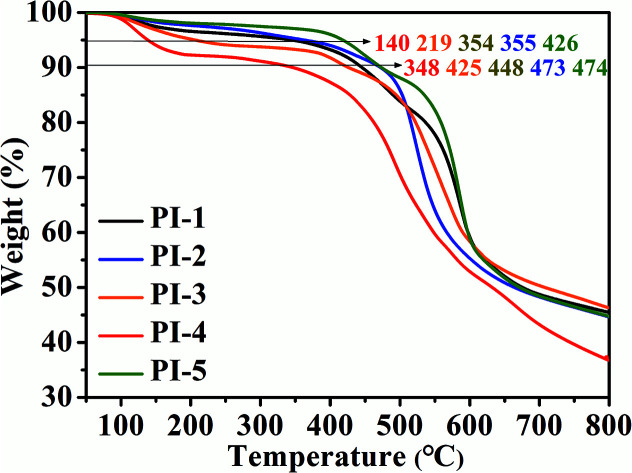

## 1. Introduction

After electrochemical oxidation or reduction, the absorption or transmittance of EC materials show reversible optical changes. EC phenomena have been known for a long time, and studies of inorganic coordination compounds, transition metal oxides, and π-conjugated and organic molecule polymer films have made great strides.^1–10^ However, non-conjugated polymers such as PIs, polyamides (PAs), polyurethanes, polysilicone, dendrimers, epoxy, *etc.*, have not been studied as EC materials extensively.^11–14^ The change of color can be adjusted with the change of the redox state of polymers. Due to their excellent thermal stability and dielectric properties, PIs are a potential competitor in the EC field. However, the main disadvantages of polymers are poor solubility and poor processability, and to a large extent, it is difficult to obtain films with excellent film-forming properties and good stability.^15^ This limits the application of the EC window because it requires a high degree of transparency and colorlessness. Liou^16^ and Sun^17,18^ proposed a strategy in that PAs prepared from alkylcycloadipic anhydride can prevent the neutral colorless electron cloud from flowing effectively. Inspired by this concept, we designed and synthesized a carbazole monomer containing an alkyl branch. The alkyl group on the main chain increases the solubility of the polymer which enables realization of roll-to-roll printing film, at the same time increasing the flexibility of the film electrode. What is more, it realizes a neutral colorless state. Functional carbazole groups are widely used in organic electronic fields such as organic field-effect transistor, organic light-emitting diode, organic photovoltaic device, organic sensor and other commercial electronic devices.^19–23^ It was demonstrated that aromatic PAs and PIs containing 4-(carbazole-9-yl)triphenylamine (CzTPA) segments exhibit attractive electrochemical and EC properties.^24–26^ In other words, CzTPA introduces benzene into carbazole at the N atom position, which has good EC performance. In addition, the carbazole block with a planar structure has strong fluorescence (PL), which endows the product with EFC properties.^27^ CzTPA-based polymers exhibit high transparency in the neutral state, in contrast to EC π-conjugated polymers, which caters to the requirement of a smart window. According to the structure of carbazole, we prepared PIs by polycondensation of a diamine monomer with five kinds of anhydrides (BTA, NTD, BPTD, DDII and BPTD). The reason is that the technical applications of most PIs are limited by their high melting points or glass transition temperatures (*T*_g_), and their limited solubility in most organic solvents. In order to overcome these difficulties, we must modify the polymer structure and introduce ethyl groups in the main chain. But one of the common ways to increase the solubility and processability of PI without sacrificing high thermal stability is the introduction of bulky phenyl, naphthyl, biphenyl, ether linkages and carbonyl groups into the polymer backbone. Furthermore, phenyl, naphthyl, biphenyl, ether linkage and carbonyl have different molecular structures, so they will play an important role in determining the electron cloud distribution in the PI backbone. In view of easy access and modulating the HOMO, LUMO and *E*_g_, different structures are selected. The solubility, thermal stability, electrochemical and EC stability and EFC properties of CzTPA-based PIs were investigated, the results indicating that PIs have prospective application in displays or smart windows.

## 2. Experimental

### 2.1. Materials

The commercially available aromatic anhydrides BTA (98%), NTD (99%), BTD (97%), DDII (98%), and BPTD (98%) bought from TCI were used as received. Tetrahydrofuran (THF), triethylamine (TEA), sodium hydride, hydrazine hydrate, and palladium on charcoal (Pd/C) (10%) were bought from Kermel Co.; tetrabutylammonium perchlorate (TBAP), 4-fluoronitrobenzene, carbazole, and 2-bromoethylamine hydrobromide (99%, J&K) were used without being treated before use. Ethanol, *N*,*N*-dimethylacetamide (DMAc), *N*,*N*-dimethylformamide (DMF), dimethyl sulfoxide (DMSO), *N*-methyl-2-pyrrolidone (NMP), acetonitrile, and methanol were used as received from Kermel Co. They were dried overnight with calcium hydroxide and purified by vacuum distillation, and stored over 4 Å molecular sieves in a sealed bottle.

### 2.2. Synthesis of PIs

PIs (named as PI-6A, PI-6B, PI-6C, PI-6D and PI-6E) were prepared by a traditional two-step progress *via* reacting an equimolar amount of a diamine monomer (M1)^27^ with various aromatic anhydrides (M2) to form poly(amic acid)s (PAAs) (named as PAA-6A, PAA-6B, PAA-6C, PAA-6D and PAA-6E), followed by heat ring dehydration. The synthesis of PI-6A is taken as an example to illustrate the general route of synthesis. 0.5360 g (0.34 mmol) diamine (M1) solution was added to 10.0 mL of a DMAc solution of BTA (0.2070 g, 0.34 mmol). After stirring at room temperature for about 24 h, a viscous PAA-6A solution was obtained. The solid content of the PAA-6A solution was about 8 wt%. The inherent viscosity of the resulting PAA-6A was 1.56 dL g^−1^, measured in DMAc at a concentration of 0.5 dL g^−1^ at 30 °C. The PAA-6A film was prepared by drip-coating a reactive polymer solution on a glass plate which was then dried overnight in vacuum at 90 °C. PAA-6A was purified from ice methanol by precipitation and being redissolved in DMAc twice. The PI was obtained by heating the PAA-6A film at 100 °C, 200 °C and 280 °C successively for 0.5 h under vacuum condition. The other PIs were prepared using similar methods.

#### PI-6A

**Fig. 1:**
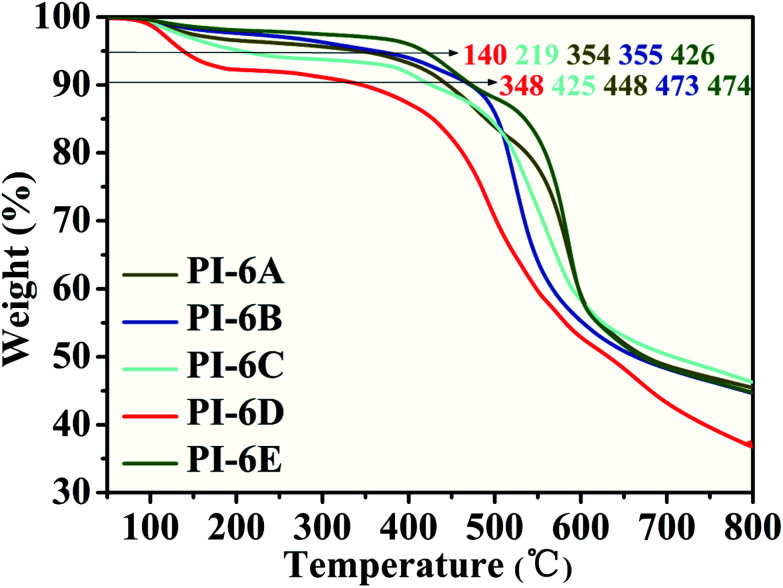
Thermogravimetric analysis curves of PI-6A–PI-6E.

**Fig. 2:**
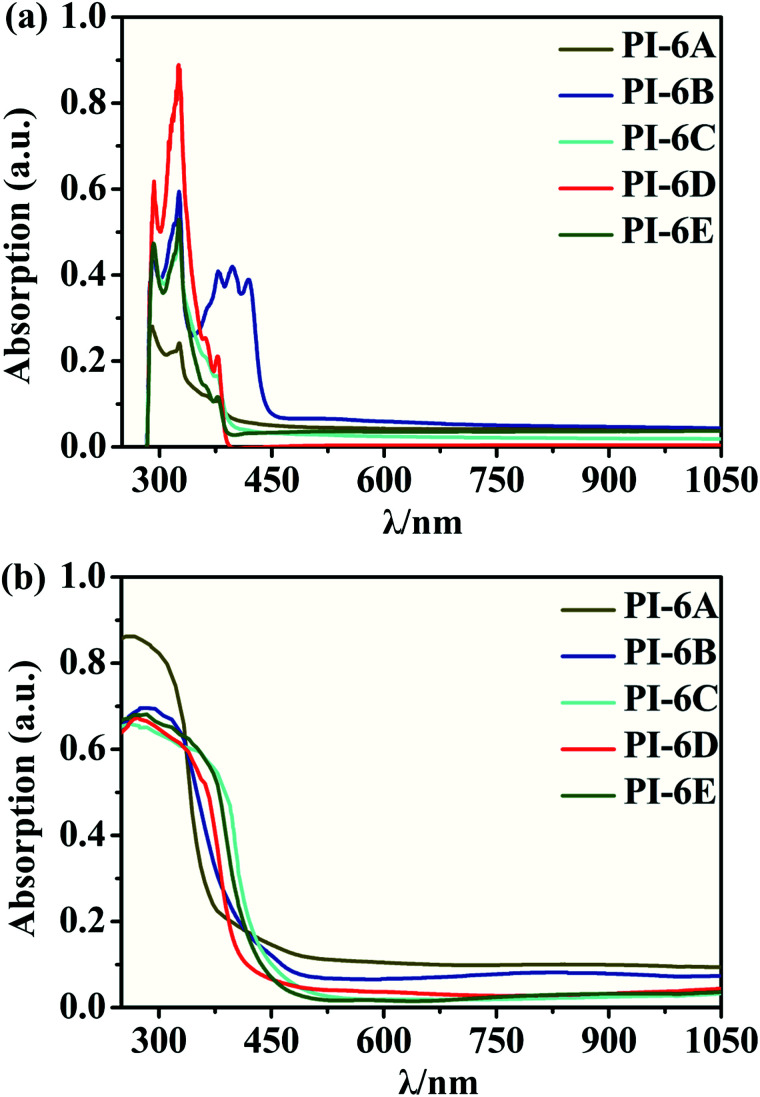
UV-visible spectra of PI-6A–PI-6E (a) in solution (solution concentration: 5 × 10 ^−5^ M) and (b) in film form.

Yield: 85%, pale yellow solid powder. FTIR (KBr, cm^−1^): 3204, 824, 752, 692 (benzene rings C–H, stretching); 2958–2863 (ethyl chains C–H, stretching); 1680, 1620 (anhydride C–O, stretching), 1180 (imide C–N, stretching) and 740 (imide C–N, bending). ^1^H NMR (400 MH_z_, DMSO-*d*_6_, *δ*, ppm): 10.5–11.0 (terminal amine groups of PAAs), 6.8–7.3 (aromatic ring of benzene) (shown in Fig. 1S and 2S†).

#### PI-6B

Yield: 81%, claybank solid powder. FTIR (KBr, cm^−1^): 3224, 824, 746, 698 (benzene rings C–H, stretching); 2928–2868 (ethyl chains C–H, stretching); 1680, 1620 (anhydride C–O, stretching), 1180 (imide C–N, stretching) and 740 (imide C–N, bending). ^1^H NMR (400 MHz, DMSO-*d*_6_, *δ*, ppm): 10.5–11.0 (terminal amine groups of PAAs), 7.3–8.2 (aromatic ring of benzene) (shown in Fig. 1S and 2S†).

#### PI-6C

Yield: 84%, red soil solid powder. FTIR (KBr, cm^−1^): 3227, 817, 746 (benzene rings C–H, stretching); 2932–2853 (ethyl chains C–H, stretching); 1680, 1620 (anhydride C–O, stretching), 1180 (imide C–N, stretching) and 740 (imide C–N, bending). ^1^H NMR (400 MHz, DMSO-*d*_6_, *δ*, ppm): 10.5–11.0 (terminal amine groups of PAAs), 7.3–8.2 (aromatic ring of benzene) (shown in Fig. 1S and 2S†).

#### PI-6D

Yield: 87%, white solid powder. FTIR (KBr, cm^−1^): 3225, 775 (benzene rings C–H, stretching); 2930–2858 (ethyl chains C–H, stretching); 1680, 1620 (anhydride C–O, stretching), 1180 (imide C–N, stretching) and 740 (imide C–N, bending). ^1^H NMR (400 MHz, DMSO-*d*_6_, *δ*, ppm): 10.5–11.0 (terminal amine groups of PAAs), 7.3–8.2 (aromatic ring of benzene) (shown in Fig. 1S and 2S†).

#### PI-6E

Yield: 83%, pale yellow solid powder. FTIR (KBr, cm^−1^): 3225, 775 (benzene rings C–H, stretching); 2950–2863 (ethyl chains C–H, stretching); 1680, 1620 (anhydride C–O, stretching), 1180 (imide C–N, stretching) and 740 (imide C–N, bending). ^1^H NMR (400 MHz, DMSO-*d*_6_, *δ*, ppm): 10.5–11.0 (terminal amine groups of PAAs), 7.3–8.2 (aromatic ring of benzene) (shown in Fig. 1S and 2S†).

Comparison of the FTIR spectra of PAAs and PIs with M1 shows that there are amide groups in PAAs and the amino groups in PIs completely disappear (shown in Fig. 1S(a) and (b)†).

Comparison of the ^1^H NMR spectra of PAAs and PIs shows that there are amide groups in PAAs and the amino groups in PIs completely disappear (shown in Fig. 2S(a) and (b)†).

### 2.3. Preparation of electrochromic device (ECD)

We fabricated a simple ECD to further study the EC properties of the PIs. A 3 mg sample of PIs (PI-6A–PI-6E) was dissolved in 1 mL of DMAc. The mixture was placed on a spin coater and spin-coated onto 1.0 cm × 4.0 cm ITO at a speed of 500 rpm to obtain a uniform film, and followed by drying in vacuum at 80 °C for 12 h. UV curing adhesive was used to seal the devices. Finally electrolyte solution (0.1 M TBAP/CH_3_CN) was injected into interlayers between two ITO glass layers. The device with PI used as electroactive material was assembled in a glovebox filled with Ar (concentration of O_2_ < 90 ppm and H_2_O < 13 ppm) and the electrolyte free of water was bubbled with N_2_ for 2 min. The testing process was carried out in sealed conditions.

## 3. Results and discussion

### 3.1. Fundamental characteristics

**Scheme 1:**
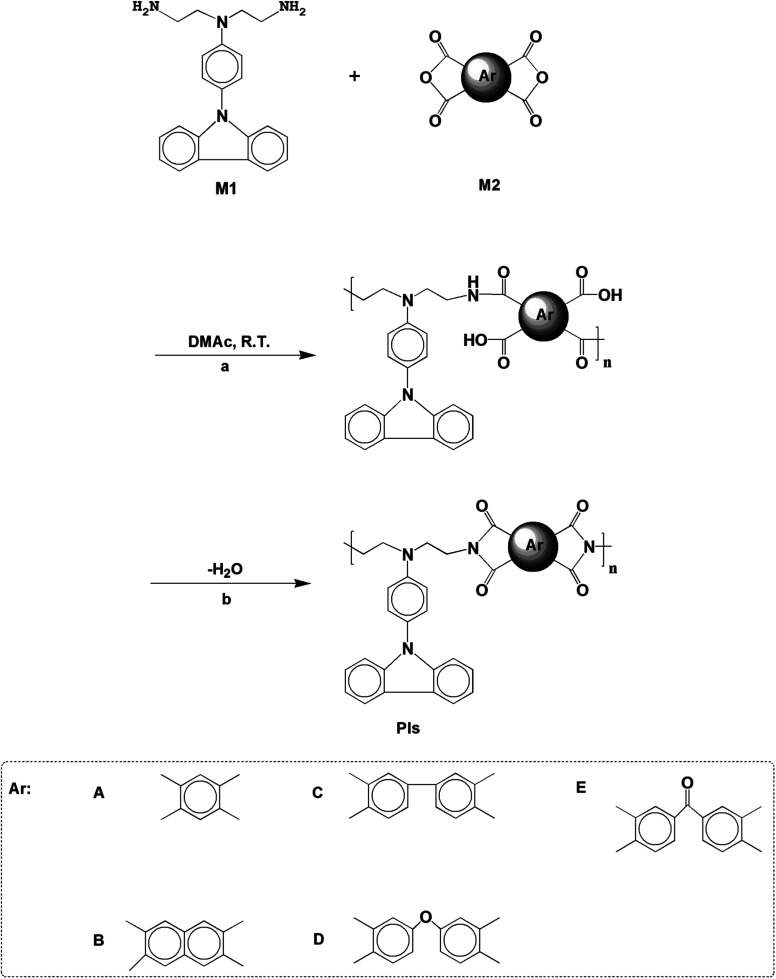
Synthetic routes of PI-6A, PI-6B, PI-6C, PI-6D and PI-6E. (a) Stirred at room temperature for about 24 hours; (b) heated in vacuum at 100 °C for 0.5 h, 180 °C for 0.5 h, then 280 °C for 0.5 h.

**Scheme 2:**
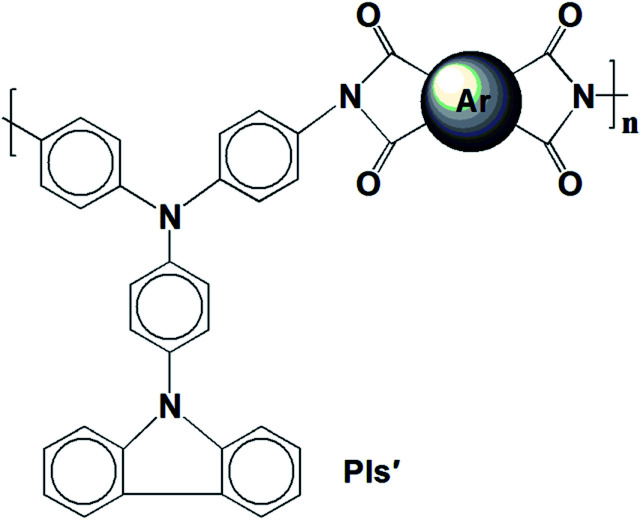
Molecular structures of analogs PI-6A′–PI-6E′.

A traditional two-step method was adopted to form PA intermediates by reacting equimolar diamine (M1) with various aromatic dianhydrides and conducting thermal dehydration or chemical ring dehydration to form PIs (PI-6A–PI-6E) (Scheme 1).^28^ We used high-temperature stage heating to cast these PA acid intermediates into flexible films, which were then converted into tough polymer films. As an alternative method, PA acid intermediates were dehydrated with a small amount of pyridine or triethylamine, and then the PIs were also formed by chemical ring cyclodehydration reaction. The complete imidization of the PIs is confirmed using the infrared spectroscopy technique. All of the PIs exhibit characteristic imide ring absorption bands near 1680, 1620 (anhydride C–O, stretching), 1180 (imide C–N, stretching) and 740 cm^−1^ (imide C–N, bending) (shown in Fig. 1S and 2S†). The absence of characteristic absorption bands for amido and carboxylic groups indicates that PIs have been fully imidized. For further research, we compared them with a series of previously reported PIs, PI-6A′–PI-6E′, which were based on 4,4′-diamino-4′′-(carbazol-9-yl)triphenylamine and dianhydrides A–E (shown in Scheme 2).^29^

Table 1 summarizes the solubility of PAAs and PIs in several organic solvents at a concentration of 10 mg mL^−1^. It can be concluded that PI samples have excellent solubility, all of which are prepared by the thermal imidization method, and are insoluble in acetonitrile, but have good solubility in other common organic solvents. When compared with the analogous PI-6A′–PI-6E′, the solubility of the 6 series of PIs has been improved, which may be due to be increased conformational flexibility or the introduction of free volume of a flexible alkyl backbone in the repeating unit instead of a benzene ring. Tough films can be obtained by thermal imination of PAA films.

**Table 1:** Solubility behavior of PAAs and PIs

Polymer code	*η*_inh_ a (dL g^−1^)	Color of solution	Solubility in various solvents^b^					
			CH_3_CN	DMAc	DMF	DMSO	NMP	THF
PAA-6A	1.56	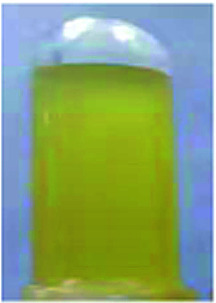	− −	+ −	+ −	+ −	+ −	+ −
PAA-6B	1.43	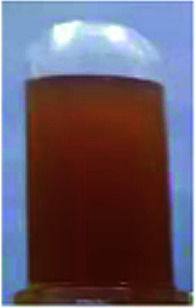	− −	+ −	+ −	+ −	+ −	+ −
PAA-6C	1.40	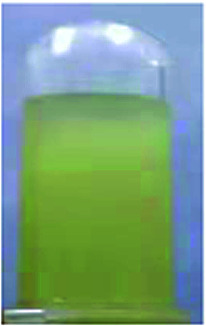	− −	+ −	+ −	+ −	+ −	+ −
PAA-6D	1.36	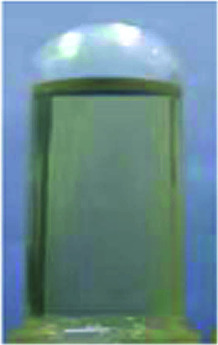	− −	+ −	+ −	+ −	+ −	+ −
PAA-6E	1.67	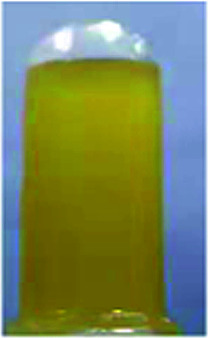	− −	+ −	+ −	+ −	+ −	+ −
PI-6A	1.03	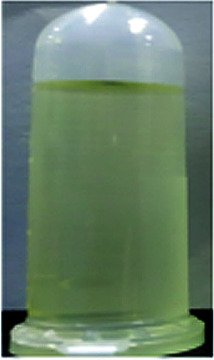	− −	+ +	+ +	+ +	+ +	+ +
PI-6B	0.81	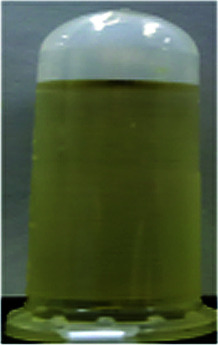	− −	+ +	+ +	+ +	+ +	+ +
PI-6C	0.82	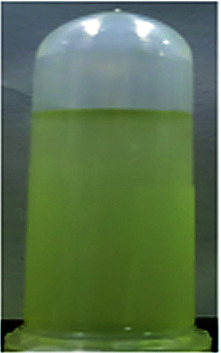	− −	+ −	+ −	+ −	+ +	+ +
PI-6D	0.87	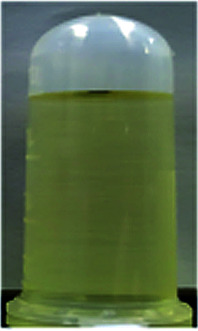	− −	+ +	+ +	+ +	+ +	+ +
PI-6E	1.19	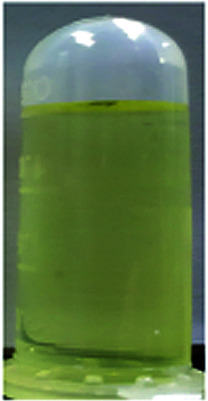	− −	+ −	+ −	+ +	+ +	+ +

a Inherent viscosity of PAAs and PIs measured at a concentration of 0.5 dL g^−1^ in DMAc at 30 °C.

b The solubility was determined at a concentration of 10 mg mL^−1^. Solubility: + +, soluble at room temperature; + −, partially soluble; − −, insoluble even on heating.

The thermal stability of PIs in nitrogen was evaluated by thermogravimetric analysis (TGA). Typical TGA curves of PIs (PI-6A–PI-6E) are shown in Fig. 1. All PIs showed good thermal stability. Under nitrogen atmosphere, the *T*_d_ values of these PIs are recorded in the range of 348–473 °C when the weight of these PIs is reduced by 10% (summarized in Table 2). From Table 2, it can be seen that the carbonization residue (carbon yield) of all PIs at 800 °C in nitrogen is in the range of 38–47 wt%. The high char yields of these PIs can be due to the presence of a large number of thermally stable aromatic hydrocarbon groups in the polymer structure. As compared to the analogous PI-6A′–PI-6E′ (640–652 °C), the introduction of alkyl groups instead of benzene rings into the polymer main chains results in a slight decrease in thermal stability.

**Table 2:** Thermal properties and molecular weights of PI-6A–PI-6E

Polymer code	*T*_d_ (°C) in N_2_			Char yield^b^ (wt%)	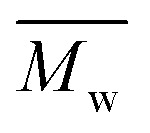 10^4^/Da	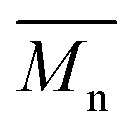 10^4^/Da	PDI^c^	PAAs		
	At 5 wt% loss^a^	At 10 wt% loss^a^	At 20 wt% loss^a^					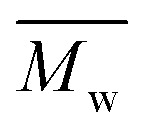 10^4^/Da	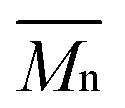 10^4^/Da	PDI^c^
PI-6A	354	448 (640)^d^	548	46	4.5	3.6	1.3	4.7	3.8	1.2
PI-6B	355	473 (—)	512	46	3.6	3.2	1.2	3.7	3.6	1.1
PI-6C	219	425 (652)	525	47	2.8	2.1	1.3	3.4	2.1	1.6
PI-6D	140	348 (648)	463	38	1.8	1.2	1.5	2.0	1.4	1.4
PI-6E	426	425 (642)	560	45	5.2	4.9	1.1	5.3	5.0	1.1

a Decomposition temperature, recorded by TGA at 10 °C min heating rate.

b Residual weight percentage at 800 °C in nitrogen.

c
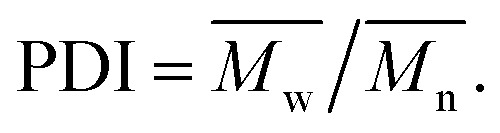

d Values in parentheses are data for analogous PI-6A′–PI-6E′.

### 3.2. Optical properties

**Fig. 3:**
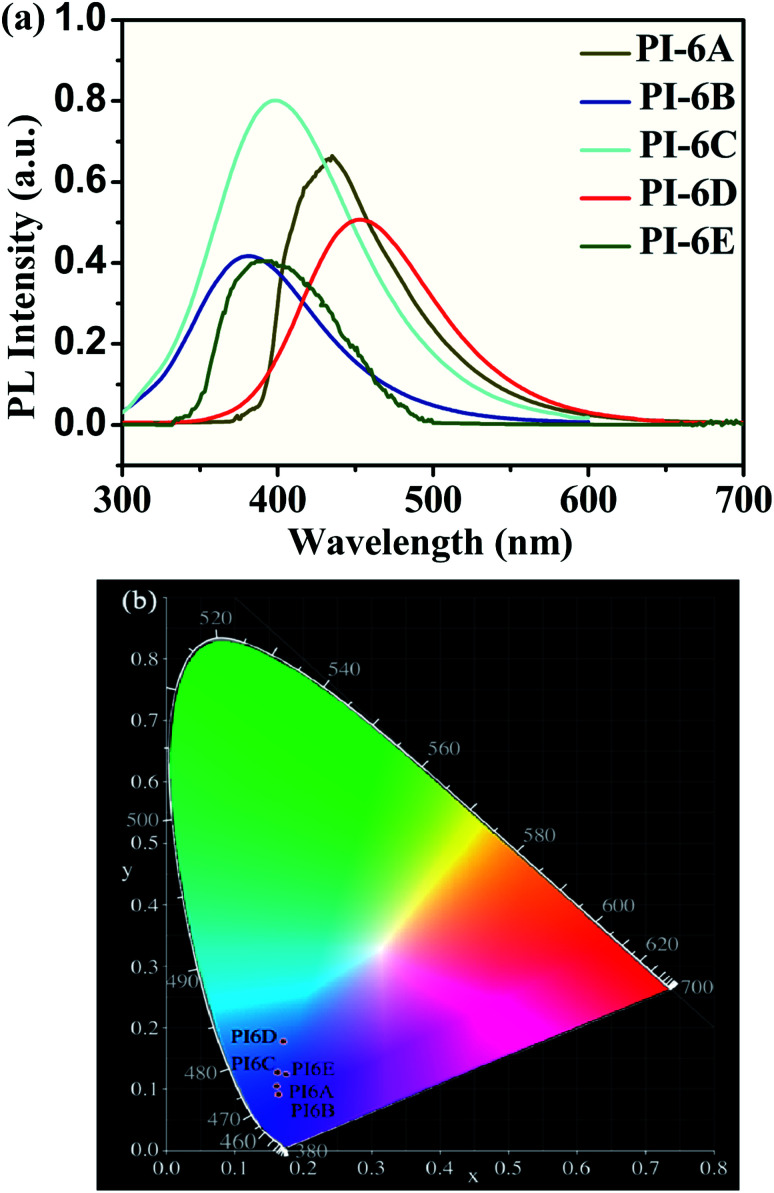
(a) PL spectra of polymers in NMP (solution concentration: 5 × 10^−5^ M) at room temperature. (b) The CIE 1931 (*x*, *y*) chromaticity diagram of PI-6A–PI-6E.

The optical properties of the PIs in NMP solution (concentration: 5 × 10^−5^ M) were studied by UV-visible spectrophotometry and PL spectroscopy (Fig. 2). The PIs show maximum absorption at 324–327 nm in NMP solution, while the absorption peaks of the PI films were at 259–284 nm, which can be mainly due to the π–π* transition of the carbon–carbon double bond in the Cz unit. However, the absorption spectra of PI films are blue-shifted compared to solution spectra. This suggests that the PIs exhibit reduced molecular planarity in the thin-film state compared to solution. In solid film, the molecules get closer than in solution due to the D–A charge effect. So we speculate that the solvent will interact with the molecules which would induce a HOMO decrease or LUMO increase resulting in the absorption spectra of PI film being blue-shifted compared to solution. The absorption values determined in this paper are similar to those reported in the literature.^30,31^ PIs show blue emission with a maximum between 382 and 453 nm at 365 nm excitation in NMP. Meanwhile, the order of the PL quantum efficiencies is PI-6C (46.3%) > PI-6D (27.1%) > PI-6B (22.8%) > PI-6A (13.8%) > PI-6E (6.4%), which are caused by the rigidity of the diamine components and the charge transfer absorption of the Cz units in the PIs, as shown in Fig. 3. PI-6C has the highest fluorescence intensity and *Φ*_PL_ due to less quenching of the charge transfer between the CzTPA donor and the imide acceptor. The optical properties of the PIs (PI-6A–PI-6E) are summarized in Table 3.

**Table 3:** Optical properties of PI-6A–PI-6E

Polymer code	In solution^a^			As film		
	*λ* ^abs^_max_ (nm)	*λ* ^PL^_max_ (nm)	*φ*_F_ b (%)	λ^abs^_max_ (nm)	*λ* ^abs^_onset_ (nm)	*λ* ^PL^_em_ (nm)
PI-6A	326	435	13.8	267 (299)^c^	372	450
PI-6B	326	382	22.8	284 (299)	462	401
PI-6C	324	399	46.3	259 (299)	456	408
PI-6D	327	453	27.1	274 (299)	409	487
PI-6E	324	395	6.4	284 (299)	448	410

a The concentration of polymer in NMP is about 5 × 10^−5^ M to measure the UV-visible absorption.

b The calculation of fluorescence quantum yield is based on quinine sulfate (*φ*_F_ = 54.6%) as a standard reference.

c Values in parentheses are data for analogous PI-6A′–PI-6E′.

### 3.3. EC properties

**Fig. 4:**
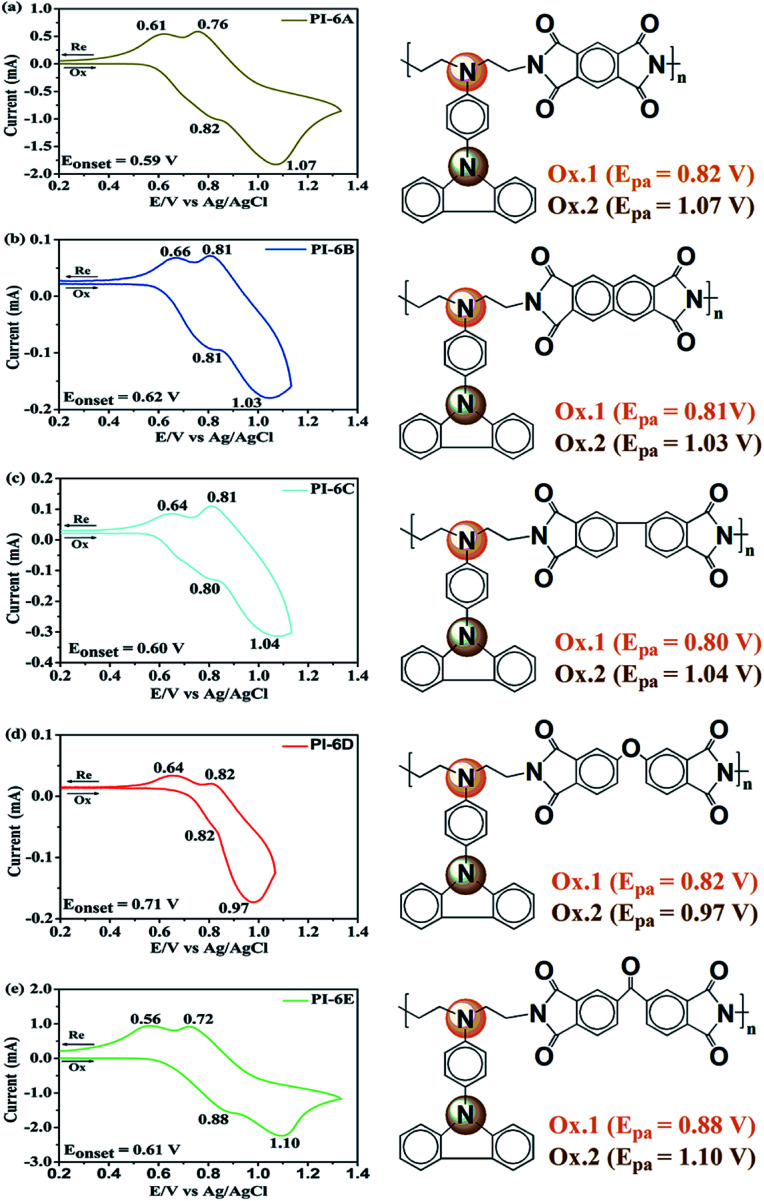
Cyclic voltammetric diagrams (left) of (a) PI-6A, (b) PI-6B, (c) PI-6C, (d) PI-6D and (e) PI-6E films on an ITO-coated glass substrate in 0.1 M TBAP/CH_3_CN solution at a scan rate of 50 mV s^−1^. The right-hand side illustrates the possible sequence of oxidation reactions in CzTPA units in PIs.

The electrochemical behaviors of PIs (PI-6A–PI-6E) were investigated using the cyclic voltammetry (CV) technique. Under an atmosphere of nitrogen, a PI film electrode was used as the working electrode. The oxidation and reduction values of polymer films were measured in CH_3_CN, as shown in Fig. 4.

Based on the potential reported by Hsiao for oxidation of these compounds,^29^ we propose a possible oxidation sequence of redox centers for PIs (PI-6A–PI-6E) (see Fig. 4). Interestingly, the TPA core may be oxidized earlier than the carbazole unit due to the electron donation of the substituent ethyl group. For example, there are double quasi-reversible oxidation redox couples at half-wave potential (*E*_1/2_) which is the average potential of the redox couple peaks, with values of 0.72 V and 0.92 V (*E*_onset_ = 0.59 V) for PI-6A (Fig. 4a). In the oxidation scan of CV of PI-6A, two spikes are observed at *E*_pa_ = 0.82 V and 1.07 V, respectively. Compared with its parent analog PI-6A′ (*E*_onset_ = 0.89 V; *E*_1/2_ = 1.05 V), PI-6A has a lower initial oxidation potential (*E*_onset_ = 0.59 V; *E*_1/2_ = 0.72 V). Earlier literature^29^ had reported that the model compound 9-phenylcarbazole exhibited a quasi-reversible redox wave at *E*_pa_ = 1.50 V. In the continuous scans, an oxidation wave at *E*_pa_ = 1.10 V gradually grows, indicative of new species formation. It can be further proved that the redox pair in potential scanning means that CzTPA^+^ participates in a very fast electrochemical reaction, resulting in a new structure that is more easily oxidized than the parent Cz. This theory was first proposed and proved by Ambrose and colleagues. In the anodization of Cz and various N-substituted Czs, ring-to-ring coupling is the main route.^32^ Coincidentally, similar results have been reported in recent publications.^33^

Other PIs show the same trend of CV behavior, and the relevant oxidation potentials are summarized in Table 4.

**Table 4:** Electrochemical properties and energy levels of PI-6A–PI-6E

Polymer code	Experimental results from CV							Quantum theoretical calculation		
	*λ*_onset_ a	*E* ^OX^_onset_ b	*E* ^OX.1^_onset_ c	*E* ^OX.2^_onset_ c	*E* ^electro^_HOMO_ d	*E* ^electro^_LUMO_ e	*E*_g_ f	*E* ^quantum^_HOMO_ g	*E* ^quantum^_LUMO_ g	*E* ^quantum^_g_ g
PI-6A	372	0.59	0.72 (1.05)^h^	0.92 (1.40)	−4.98^h^ (−5.41)	−1.65	3.33	−5.13	−2.98	2.15
PI-6B	462	0.62	0.74 (—)	0.92 (—)	−5.01 (—)	−2.42	2.59	−5.18	−3.16	2.02
PI-6C	456	0.60	0.72 (1.07)	0.93 (1.38)	−5.01 (−5.43)	−2.29	2.72	−5.18	−2.91	2.27
PI-6D	409	0.71	0.73 (1.06)	0.90 (1.36)	−5.05 (−5.42)	−2.02	3.03	−4.99	−3.09	1.90
PI-6E	448	0.61	0.72 (1.08)	0.91 (1.40)	−5.12 (−5.44)	−2.34	2.78	−5.19	−3.25	1.94

a UV-visible absorption starting wavelength.

b The onset oxidation potential *vs.* Ag/AgCl.

c *E*_1/2_ is average potential of the redox couple peaks.

d *E*_HOMO_ (eV) = −e(*E*_ox/onset_*vs.* Ag/AgCl + 4.43) eV; HOMO is the highest occupied molecular orbital.

e *E*_LUMO_ (eV) = *E*_HOMO_ + *E*_g_; LUMO is lowest unoccupied molecular orbital.

f *E*_g_ (eV) = 1240/*λ*_onset_.

g Values of theoretical calculation.

h Values in parentheses are data for analogous PI-6A′–PI-6E′.

**Fig. 5:**
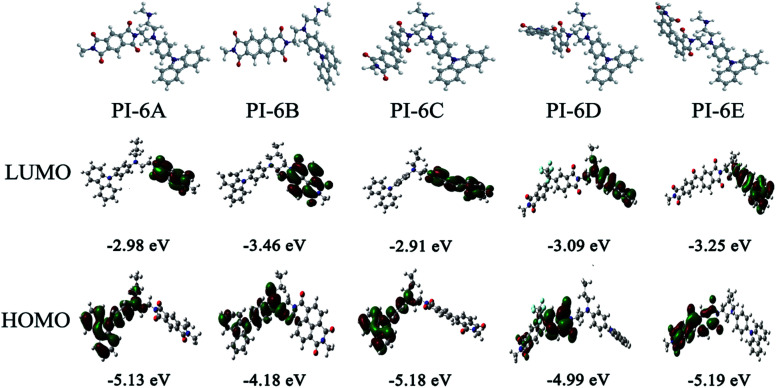
Graphical representation of the electron density in the frontier molecular orbitals of the repeating units of PI-6A–PI-6E.

The redox potentials of the various PIs (PI-6A–PI-6E) are summarized in Table 4, and HOMO and LUMO potentials of the PIs are shown in Fig. 5. The HOMO levels of the PIs are evaluated as −4.98 to −5.12 eV and −4.99 to −5.19 eV, calculated from the experimental results of CV (assuming that the HOMO level of ferrocene is 4.43 eV compared with the zero vacuum level) and from quantum theoretical calculation, respectively. And the HOMO levels of the analogous PI-6A′–PI-6E′ were reported as −5.41 to −5.44 eV in the literature. The HOMO energy levels of PIs are all close to the air oxidation threshold (−5.27 eV),^34^ which indicates that the synthesized PIs have good air oxidation resistance. Due to the quantization calculation of the individual units of the PIs,^35^ and the differences in solvent effects and measurement methods,^36^ the results show that the calculated values agree well with the optical experimental data. These data are used to calculate the LUMO energy level. The low ionization potential indicates that it is easier for ITO electrodes to inject holes into active films in electronic devices.

**Fig. 6:**
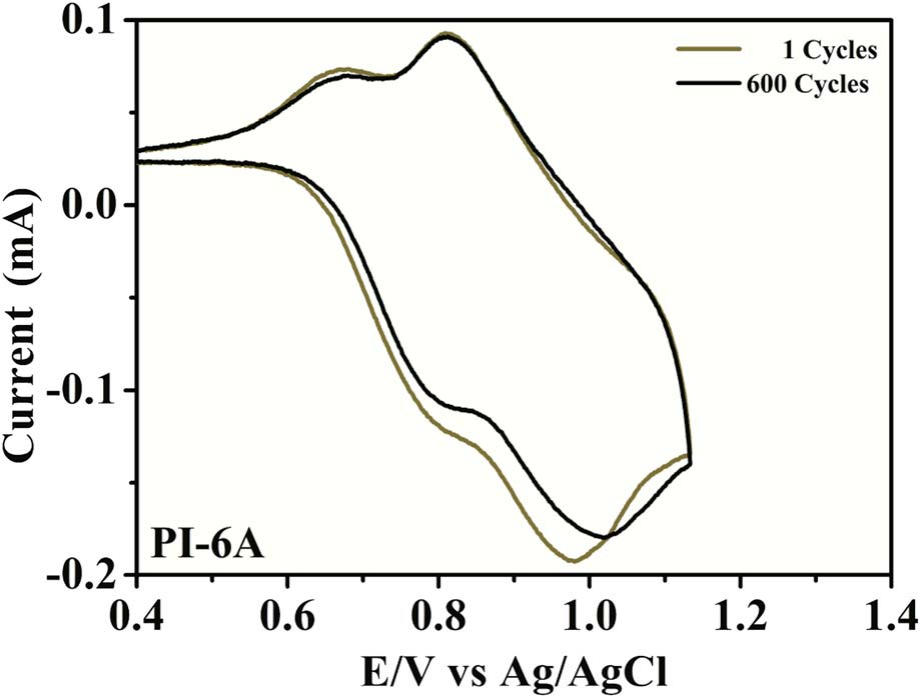
Electrochemical stability of PI-6A film on an ITO-coated glass substrate in 0.1 M TBAP/CH_3_CN solution at a scan rate of 50 mV s^−1^ after 600 switching cycles determined by the CV method.

To further investigate the redox stability of PI material, CV tests were conducted of the PI films deposited on ITO electrodes using potential scan between neutral and oxidation states in 0.1 M TBAP/CH_3_CN without monomer at a potential scan rate of 50 mV s^−1^, as shown in Fig. 6. Retained electroactivity was observed for PI-6A (92%) after 600 cycles. A similar trend is observed for the other PIs (shown in Fig. S3†). The retained electroactivities were observed to be 94% (PI-6B), 91% (PI-6C), 88% (PI-6D) and 99% (PI-6E) after 600 cycles. Since the PIs are in direct contact with the electrolyte, degradation reaction occurs under the catalysis of trace oxygen and water. If the electrodes are assembled into an ECD being sealed off from the air, the switch stability will be greatly improved. Overall, the PIs are both very robust and redox stable, making them good candidates for EC applications.

**Fig. 7:**
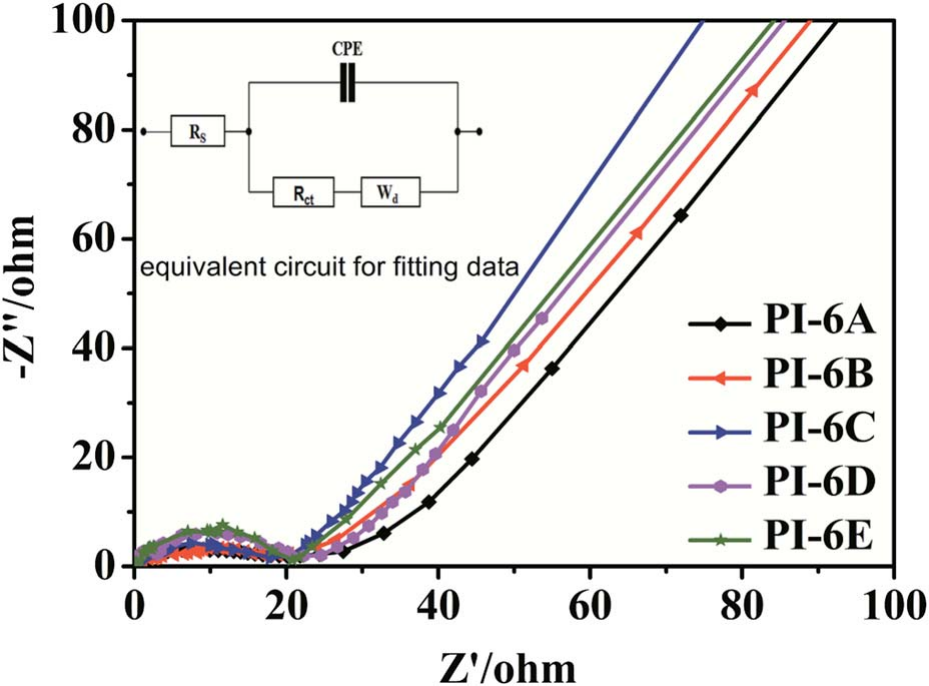
Impedance spectra of PI-6A–PI-6E films on an ITO-coated glass substrate.

The electrical resistances between the PIs and the electrolyte were further evaluated by electrochemical impedance spectroscopy (EIS) and fitted by Zview software.^37^Fig. 7 shows impendence spectra of PI-6A–PI-6E films. CPE is the capacitance between the substrate and the PI film, and *R*_s_ is the series resistance. Warburg diffusion element (*W*_d_) is a commonly used diffusion circuit element that simulates semi-infinite linear diffusion, that is, unlimited diffusion of large planar electrodes.^38–41^*W*_d_ is difficult to identify because it is always associated with double layer capacitance and charge-transfer resistance. The charge-transfer resistance *R*_ct_ is the resistance when a current passes through one electron or ion in the system and causes a voltage difference between the other electrons and ions in the system, and the charge transfer can be known by the transfer impedance (it is difficult for electrons and ions to undergo electrochemical reactions at the electrode/electrolyte interface to transfer to the surface of the active material).^42^ Among the above parameters, *R*_ct_ is the most critical parameter affecting EC performance. PI-6B has the lowest *R*_ct_ value among the five polymers, indicating that ions migrate faster between the PI film and electrolyte. However, the *R*_ct_ of PI-6D with the highest charge and ion transfer resistance is 20.85 Ω. The reason for the poor ion transfer capacity may be the poor charge and ion transfer capacity of the PI-6D film. Table 5 summarizes the data obtained by fitting the equivalent circuit. The *R*_ct_ for conjugated polymers reported in the literature is in the range 15–35 Ω. The *R*_ct_ values of PIs in this article are similar to those reported in the literature.^43,47^

**Table 5:** Fitting values of the equivalent circuit elements of PI-6A–PI-6E

Polymer code	Equivalent circuit elements		
	*R*_s_ (Ω)	CPE-T	*R*_ct_ (Ω)
PI-6A	4.1	1.10 × 10^−4^	18.23
PI-6B	5.6	1.42 × 10^−4^	17.60
PI-6C	5.7	1.48 × 10^−4^	18.71
PI-6D	4.5	1.74 × 10^−4^	20.85
PI-6E	9.6	2.79 × 10^−4^	20.74

**Fig. 8:**
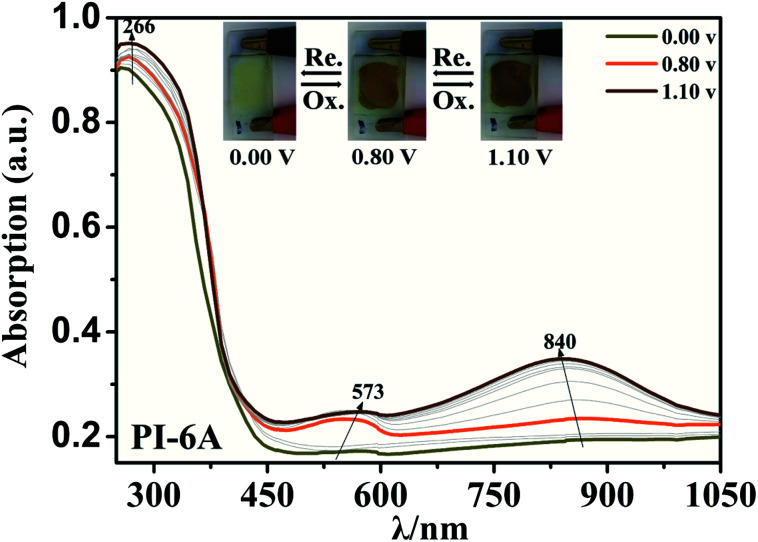
Absorption changes of PI-6A film on ITO-coated glass substrate (V *vs.* Ag/AgCl) with 0.1 M TBAP/CH_3_CN solution as supporting electrolyte. The inset shows the color change of the film at the indicated applied voltage.

Optical properties changed after oxidation by EC experiments. The absorption values are similar to those for the analog PI-6A′ reported in the literature. PI-6A′ showed two strong absorption peaks at 296 and 330 nm in the neutral form. Under oxidation (the applied voltage increased from 0.0 V to 1.1 V), there was a new peak at 412 nm, and the near-infrared region expanded from 800 nm to 1100 nm, and the intensity gradually increased. In comparison, the spectral changes of PI-6A in different oxidation states are shown in Fig. 8. In neutral form, at 0 V (*vs.* Ag/AgCl), PI-6A exhibits a strong absorption peak at approximately 266 nm, representing a characteristic π–π* transition, which is almost transparent. When the PI-6A film is oxidized with the applied voltage being increased from 0.00 V to 0.80 V, the absorption peak intensity increases gradually at 340 nm. At the same time, a new absorption peak appears at 573 nm, the intensity of which gradually increases. We attribute these spectral changes to the formation of localized CzTPA electronic stability. As the applied potential increases toward 1.10 V, the number of cationic radicals increases gradually. A new strong absorption band is formed in the near-infrared region, and its center position is around 840 nm which is attributed to the formation of cations in the CzTPA segment. The UV-visible absorption changes of PI-6A films at different potentials are completely reversible which are easily seen by the naked eye. We can observe from Fig. 8 that the PI-6A film changes from a light yellow transparent neutral state to a brown yellow highly absorbing semi-oxidized state and a deep yellow fully oxidized state. Although the color is basically in the ultraviolet absorption region, but because there is no conjugate channel, there is no strong electron cloud shift, so the color of the film is almost colorless. Other PIs have similar trends (as shown in Fig. S4†). Meanwhile, the typical spectral electrochemical and transmission-wavelength application potential correlations of PI-6A–PI-6E films are shown in Fig. S5.†

**Fig. 9:**
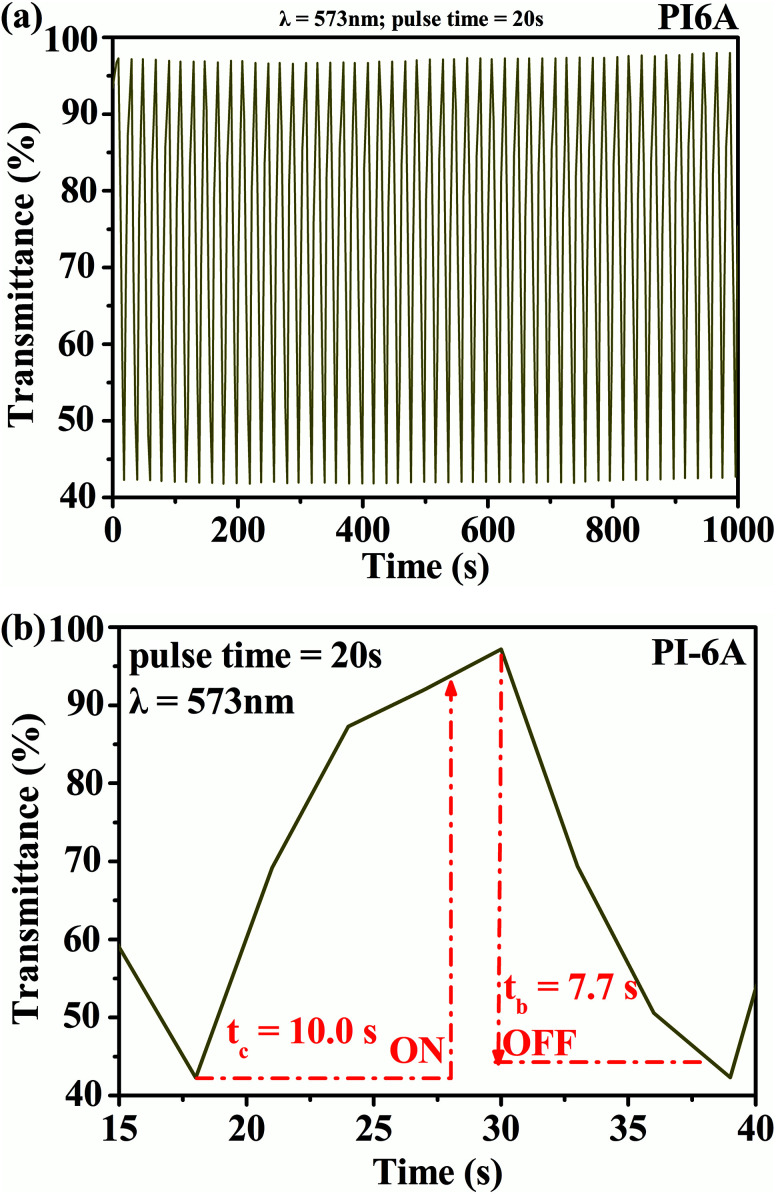
(a) Square-wave potential step absorptiometry and (b) optical switching for PI-6A film (monitored at 573 nm).

The square wave potential method was used to study the optical response characteristics of PI (PI-6A–PI-6E) films. The changes in transmittance of the films were measured and recorded at the maximum absorption wavelength by periodically converting the voltages in the neutral and oxidation states at certain time intervals. From Fig. 9, it can be concluded that the transmittance of the PI-6A film at a maximum incident wavelength time interval of 20 s at 573 nm is 55%. In addition, compared with some reported polymers with similar backbones,^44^ the PI-6A film in this paper has higher optical contrast. However, when the time interval is set to 20 s, the fading time of PI-6A from the oxidized state to the reduced state is longer, lasting 10.0 s. The corresponding coloring time from the neutral state to the oxidized state is 7.7 s. Table 6 summarizes the optical contrast, response time, and coloring efficiency of the PI films at the maximum absorption wavelength in a time interval of 20 s.

**Table 6:** EC properties of PI-6A–PI-6E

Polymer code	*λ* a, nm	Δ*T*, %	Response time^b^		ΔOD^c^	*Q*_d_ d, mC cm^−2^	CE^e^, cm^2^ C^−1^
			*t*_c_ (s)	*t*_b_ (s)			
PI-6A	573	55	10.0	7.7	0.365	1.91	191
PI-6B	455	25	7.5	6.0	0.129	2.09	62
PI-6C	543	53	9.0	7.5	0.376	1.98	190
PI-6D	568	57	8.5	5.5	0.477	1.91	250
PI-6E	554	45	10.0	8.0	0.327	1.93	169

a The specified wavelength of measurement data.

b Voltage application time required to reach 90% of maximum transmittance at specified wavelength.

c ΔOD = log(*T*_b_/*T*_c_), where *T*_c_ is the maximum transmittance in colored state and *T*_b_ the maximum transmittance in faded state.

d *Q*_d_ is the number of charges in or drawn out per unit area, determined from the *in situ* experiments.

e Coloration efficiency is the ratio of the change in the optical absorption of a material to the charge and loss per unit area at a specified wavelength, derived from the equation CE = ΔOD/*Q*_d_.

The response speed of PI-6A film is similar to that of some other soluble EC PI films reported in the literature,^45,46^ which is enough to be comparable to that of EC conductive polymers. However, the advantages of absorption contrast and response time make it possible to develop and apply in non-light-emitting display devices. Other PIs (PI-6A–PI-6E) have similar trends (shown in Fig. S6†).

### 3.4. EFC properties

**Fig. 10:**
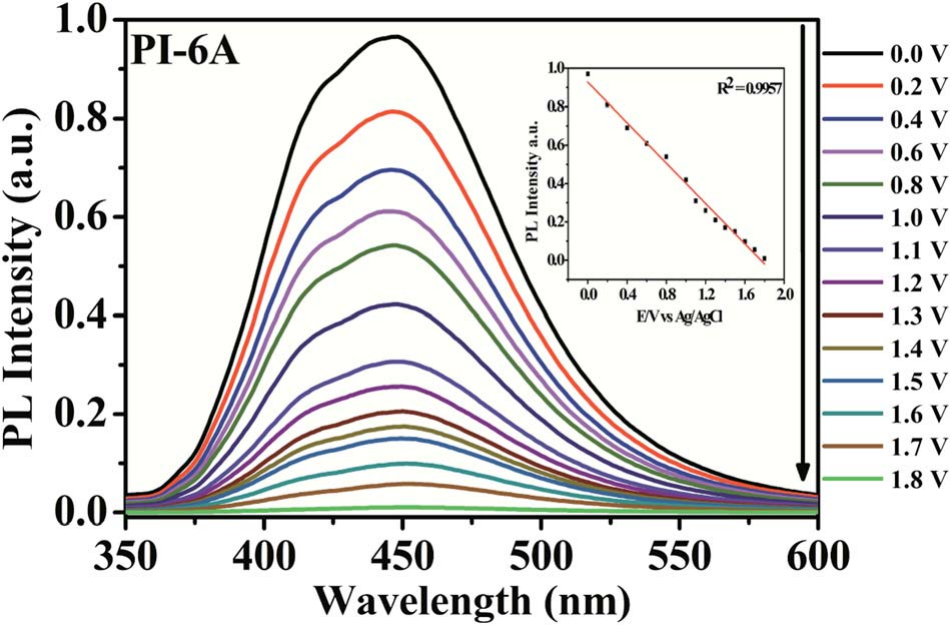
PL spectral changes of PI-6A film upon application of different applied potentials.

It is worth noting that PI films have not only EC behavior but also EFC behavior. As shown in Fig. 10, upon application of a series of positive potentials, PI-6A shows its EFC switch characteristics. Upon being excited at 365 nm, dynamic response behavior was tested through an oxidation step of 0.0 to 1.8 V. When the voltage increases, the PL intensity of PI-6A decreases which is attributed to the increase of the effective PL quencher (CzTPA^+^) number in the polymer. At the same time, the intensity of the maximum PL peak of neutral PI-6A at 448 nm decreases significantly and continuously with the increase of applied potential. When the voltage is as high as 1.8 V, the PL almost disappears and the PL spectrum is close to the baseline. In comparison to PI-6A, fluorescence of other PIs is quenched by increasing the voltage above 2.0 V or 1.8 V (shown in Fig. S7†). The PL of PIs indicates that the fluorescence quenching originated from the electrochemical oxidization of the PIs.^47,48^

## 4. Conclusion

A series of novel PIs with EC and EFC properties containing flexible carbazole blocks were synthesized by the traditional two-step method. All of the prepared PI films have excellent thermal stability. The oxidation potentials of the polymers are significantly reduced. These PIs also show good electrochemical stability, and retention of electroactivities is observed to be 92% (PI-6A), 94% (PI-6B), 91% (PI-6C), 88% (PI-6D) and 99% (PI-6E) after 600 cycles. After two-stage oxidation, the color changes from yellowish to neutral brown yellow and dark yellow in the oxidized state. In addition, PIs show excellent and unique optical behavior in both solution and film states, and have different emission colors and PL quantum yields. Therefore, the application and development prospects of prefabricated ECDs can be greatly improved.

## Conflicts of interest

There are no conflicts to declare.

## Supplementary Material

RA-010-C9RA10515H-s001

## References

[cit1] Monk P. M. S. , Mortimer R. J. and Rosseinsky D. R., Electrochromism and electrochromic devices, Cambridge University Press, New York, 2007

[cit2] Qiu B. W., Li Z. J., Wang X., Li X. Y., Zhang J. R. (2017). J. Polym. Sci., Part A: Polym. Chem..

[cit3] Chen K. Y., Lai Y. S., You J. K., Santiago K. S., Yeh J. M. (2019). Polymer.

[cit4] He J., Chen F., Lindsay S. (2007). Appl. Phys. Lett..

[cit5] Zhou Y., Liu X. C., Jia X. T., M Chao D. (2019). New J. Chem..

[cit6] M Chen Q., J Qian X., Xu Y. S., Yang Y., Wei Y., Ji Y. (2019). ACS Appl. Mater. Interfaces.

[cit7] Apparao T., Karteek B., Ramanuj N., Sreedhar B., Chepuri R. K. R. (2019). J. Electroanal. Chem..

[cit8] Liu T., Wang R., Dong Z. F., Zhu Z. G., Zhang X. Q., Liu J. G. (2018). J. Appl. Polym. Sci..

[cit9] Ye Y. W., Zhang D. W., Liu T., Liu Z. Y., Liu W., Pu J. B., Chen H., Zhao H. C. (2019). J. Hazard. Mater..

[cit10] Hung W. I., Hung C. B., Chang Y. H., Dai J. K., Li Y., He H., Chen S. W., Huang T. C. (2011). J. M. J. Mater. Chem..

[cit11] Yang C. Y., Cai W. A., Zhang X., Gao L. X., Lu Q. Y., Niu H. J., Wang W. (2019). Dyes Pigm..

[cit12] Yen H. J., Liou G. S. (2009). Chem. Mater..

[cit13] Chuang Y. W., Yen H. J., Wu J. H., Liou G. S. (2014). ACS Appl. Mater. Interfaces.

[cit14] Hsiao S. H., Hsiao Y. H. (2017). High Perform. Polym..

[cit15] Santhanamoorthi N., Shie W. R., Liaw D. J., Roman V. R., Jiang J. C. (2019). J. Phys. Chem. B.

[cit16] Wu J. T., Lin H. T., Liou G. S. (2019). ACS Appl. Mater. Interfaces.

[cit17] Sun N. W., Su K. X., Zhou Z. W., Yu Y., Tian X. Z., Wang D. M., Zhao X. G., Zhou H. W., Chen C. H. (2018). ACS Appl. Mater. Interfaces.

[cit18] Sun N. W., Su K. X., Zhou Z. W., Zhou X., Franziska Lissel W., Zhao X. G., Chen C. H. (2019). Macromolecules.

[cit19] Liou G. S., Hsiao S. H., Huang N. K., Yang Y. L. (2006). Macromolecules.

[cit20] Cheng S. H., Hsiao S. H., Su T. X., Liou G. S. (2005). Macromolecules.

[cit21] Li M., Kang S. S., Du J., Zhang J., Wang J. X., Ariga K. (2018). Angew. Chem., Int. Ed..

[cit22] Zhang J., Du J., Wang J. X., Wang Y. F., Wei C., Li M. (2018). ACS Appl. Mater. Interfaces.

[cit23] Hsiao S. H., Wang H. M., Chang P. C., Kung Y. R., Lee T. M. (2013). J. Polym. Sci., Part A: Polym. Chem..

[cit24] Tsai M. H., Ke T. H., Lin H. W., Wu C. C., Chiu S. F., Fang F. C., Liao Y.-L., Wong F. T., Chen Y. H., I Wu C. (2009). ACS Appl. Mater. Interfaces.

[cit25] Tao Y. T., Wang Q., Yang C. L., Zhong C., Zhang K., Qin J. G., Ma D. G. (2010). Adv. Funct. Mater..

[cit26] Liou G. S., Hsiao S. H., Chen H. W. (2006). J. Mater. Chem..

[cit27] Sudesh M., Sudhakar M., Kalidass K., Parthasarathy V. (2019). J. Org. Chem..

[cit28] Zheng R. R., Zhang X., P Zhang Z., Niu H. J., Wang C. (2019). Appl. Surf. Sci..

[cit29] Wang H. M., Hsiao S. H. (2014). J. Polym. Sci., Part A: Polym. Chem..

[cit30] Cheng S. H., Hsiao S. H., Su T. H., Liou G.-S. (2005). Macromolecules.

[cit31] Kung Y. C., Hsiao S. H. (2011). J. Mater. Chem..

[cit32] Ambrose J. A., Carpenter L. L., Nelson R. F. (1975). J. Electrochem. Soc..

[cit33] Wang H. M., Hsiao S. H. (2014). J. Polym. Sci., Part A: Polym. Chem..

[cit34] Sista P., Nguyen H., Murphy J. W., Hao J., Dei D. K., Palaniappan K., Servello J., Kularatne R. S., Gnade B. E., Xue B., Dastoor P. C., Biewer M. C., Stefan M. C. (2010). Macromolecules.

[cit35] Lu K., Di C. A., Xi H. X., Liu Y. Q., Yu G., Qiu W. F., Zhang H. J., Gao X., Liu Y., Qi T., Du C. Y., Zhu D. B. (2008). J. Mater. Chem..

[cit36] Thompson B. C., Kim Y. G., Mccarley T. D., Reynolds J. R. (2006). J. Am. Chem. Soc..

[cit37] Zheng R. Z., Zhang J., Jia C., Wan Z., Fan Y., Weng X., Xie J., Deng L. (2017). Polym. Chem..

[cit38] Li J. F., Ge S. S., Wang J. X., Du H. Y., Song K. N., Fei Z. Y., Shao Q., Guo Z. H. (2018). Colloids Surf., A.

[cit39] Tian J. Y., Shao Q., Dong X. J., Zheng L., Pan J. D., Zhang X. Y., Cao H. L., Hao L. H., Liu J. R., Mai X. M., Guo Z. H. (2018). Electrochim. Acta.

[cit40] Guo X. K., Ge S. S., Wang J. X., Zhang X. C., Zhang T., Lin J., Zhao C. X., Wang B. (2018). Polymer.

[cit41] Wang T., Le Q., Zhang J. (2017). Electrochim. Acta.

[cit42] Qian L., Lv X., Ouyang M., Tameev A., Katin K., Maslov M., Bi Q., Huang C., Zhu R., Zhang C. (2018). ACS Appl. Mater. Interfaces.

[cit43] Lu Q. Y., Zhang X., Cai W. A., Wang Y., Niu H. J., Wang W. (2019). Sol. Energy Mater. Sol. Cells.

[cit44] Mi S., Wu J. C., Liu J., Zheng J. M., Xu C. Y. (2015). Org. Electron..

[cit45] Goker S., Hizalan G., Aktas E., Kutkan S., Cirpan A., Toppare L. (2016). New J. Chem..

[cit46] Liu J., Mi S., Xu Z. P., Wu J. C., Zheng J. M., Xu C. Y. (2016). Org. Electron..

[cit47] Sun J. W., Liang Z. Q. (2016). ACS Appl. Mater. Interfaces.

[cit48] W Sun N., Zhou Z. W., Meng S. Y., Chao D. M., Chu X. J., Zhao X. G. (2017). Dyes Pigm..

