# Supplementary material for: Novel polyimides containing flexible carbazole blocks with electrochromic and electrofluorescencechromic properties
Source: RSC Adv. 2020 Feb 14;10(12):6992–7003. doi: 10.1039/c9ra10515h (PMC9049831; doi:10.1039/c9ra10515h)
Supplement: RA-010-C9RA10515H-s001 [file RA-010-C9RA10515H-s001.pdf]

Electronic Supplementary Material (ESI) for RSC Advances.
This journal is © The Royal Society of Chemistry 2020

Appendix A. Supplementary data

S1 Instruments and measurements

Infrared spectra in the range of 4000-400 cm^{-1} were recorded on the PerkinElmer Spectrum 100 model FT-IR spectrometer. The ^{1}H NMR and ^{13}NMR spectra of tetramethylsilane were measured at Bruker AC-400 MHz and AC-100 MHz respectively. Uv-vis spectra were measured at Shimadzu UV-3600. The fluorescence spectra were recorded with JASCO FP-6200 PL spectrometer. The thermogravimetric analyses by PerkinElmer Pyris 6 thermogravimetric analyzer were carried out in nitrogen atmosphere. The inherent viscosities of the polymers were determined at 0.5 g/dL concentration using an Ubbelohde viscometer at 30 ^{o}C. The thicknesses of the PIs films were determined on a profile-system XP-100 (KLA-Tencor) by comparing them to the thickness of a blank ITO-coated glass slide. Devices were prepared to operate in glove box model JMS-1X. The experiments were conducted on about 6-8 mg powder samples heated in flowing nitrogen or air (flow rate: 20 $cm^{3}\cdot min^{-1}$) at a heating rate of 10 $^{o}C\cdot min^{-1}$. The manufacturing process and measurement of the device were performed in room temperature air. Before being used, ITO was washed by ultrasonic for 30 minutes in detergent, deionized water and dichloromethane successively. CV and spectroelectrochemical analysis were performed using a three-electrode electrochemical cell in 0.1M tetramethylammonium perchlorate (TBAP) solution at room temperature of 50 $mV\cdot s^{-1}$. Ultraviolet tube used as a holder and indium tin oxide (ITO, 1.0 cm × 4.0 cm), platinum wire and Ag/AgCl (0.1 M) are used as working electrode, the electrode and reference electrode, respectively. Theoretical studies were carried out on the 6-31G basis set in Gaussian 09 using density functional theory (DFT), which was approximated by B3LYP.

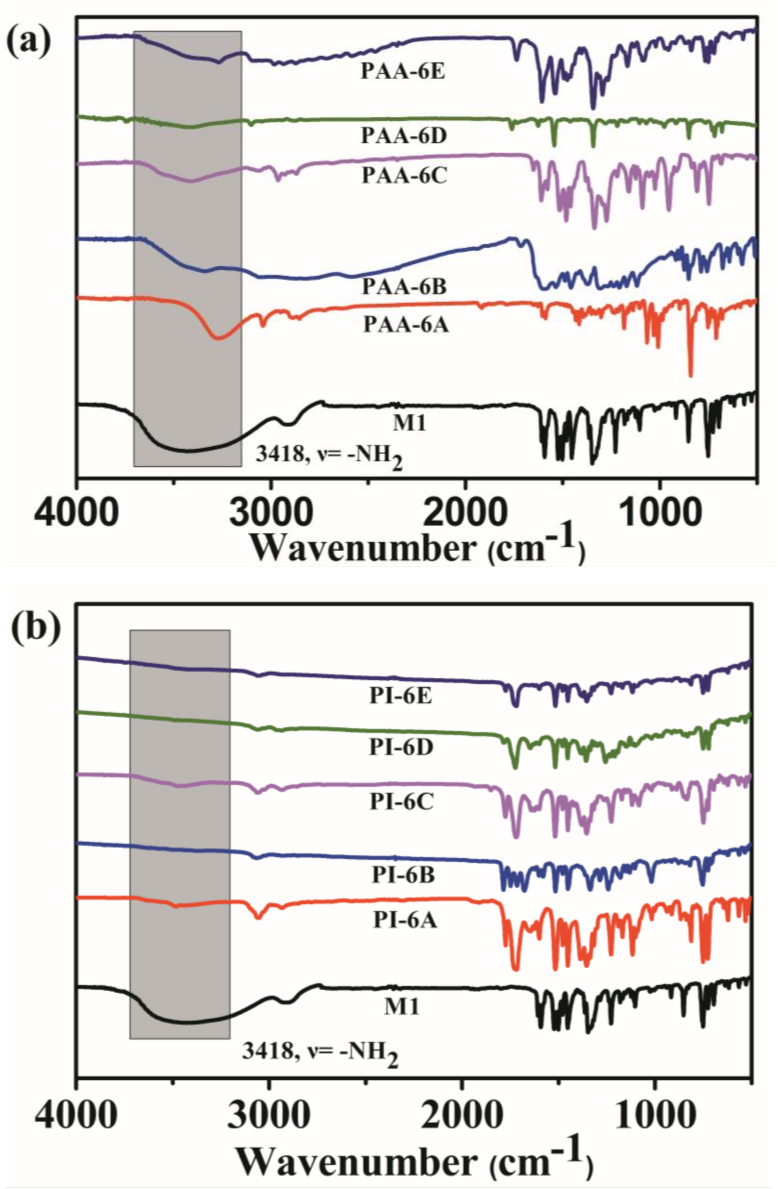

Fig. S1 FTIR spectra of (a) PAAs and (b) PIs.

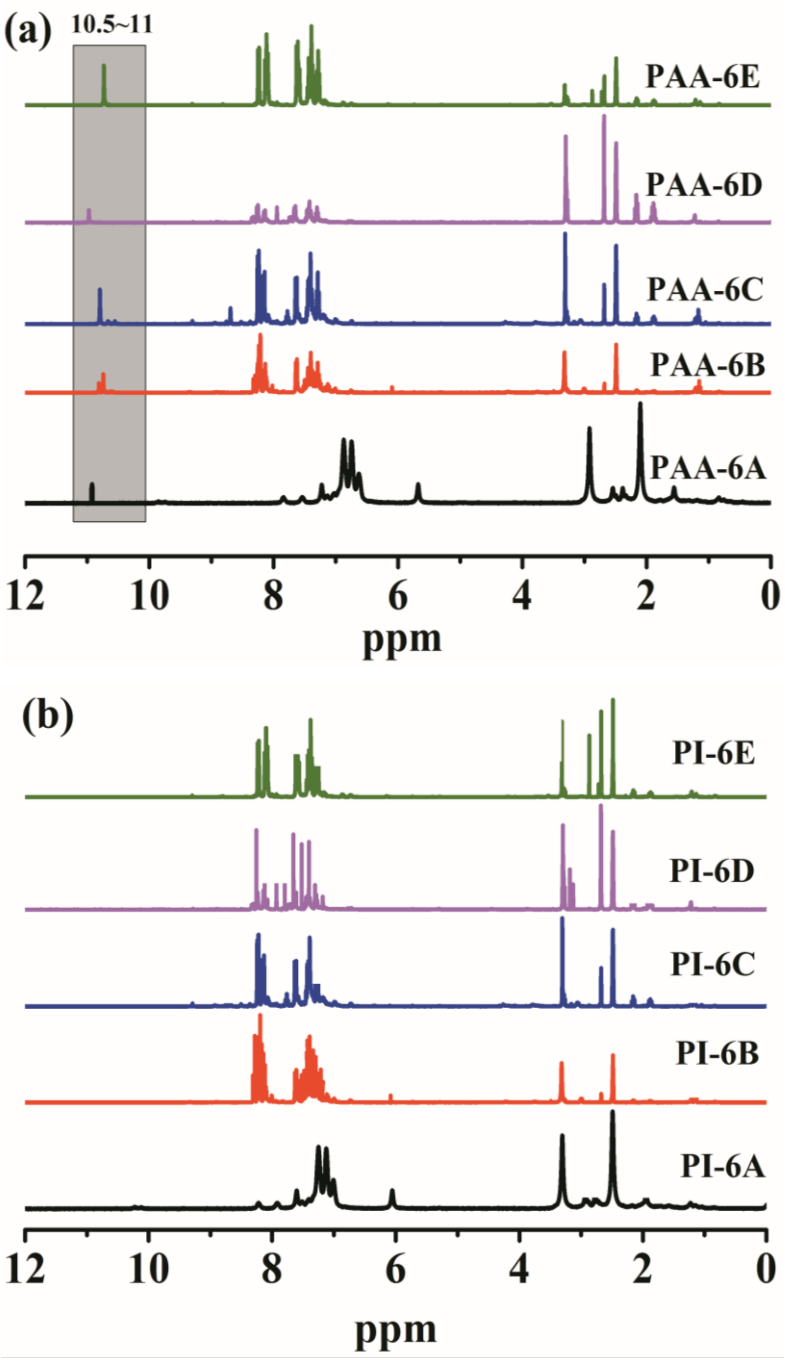

Fig. S2 ^{1}H NMR spectra of (a) PAAs and (b) PIs and in DMSO-d_6.

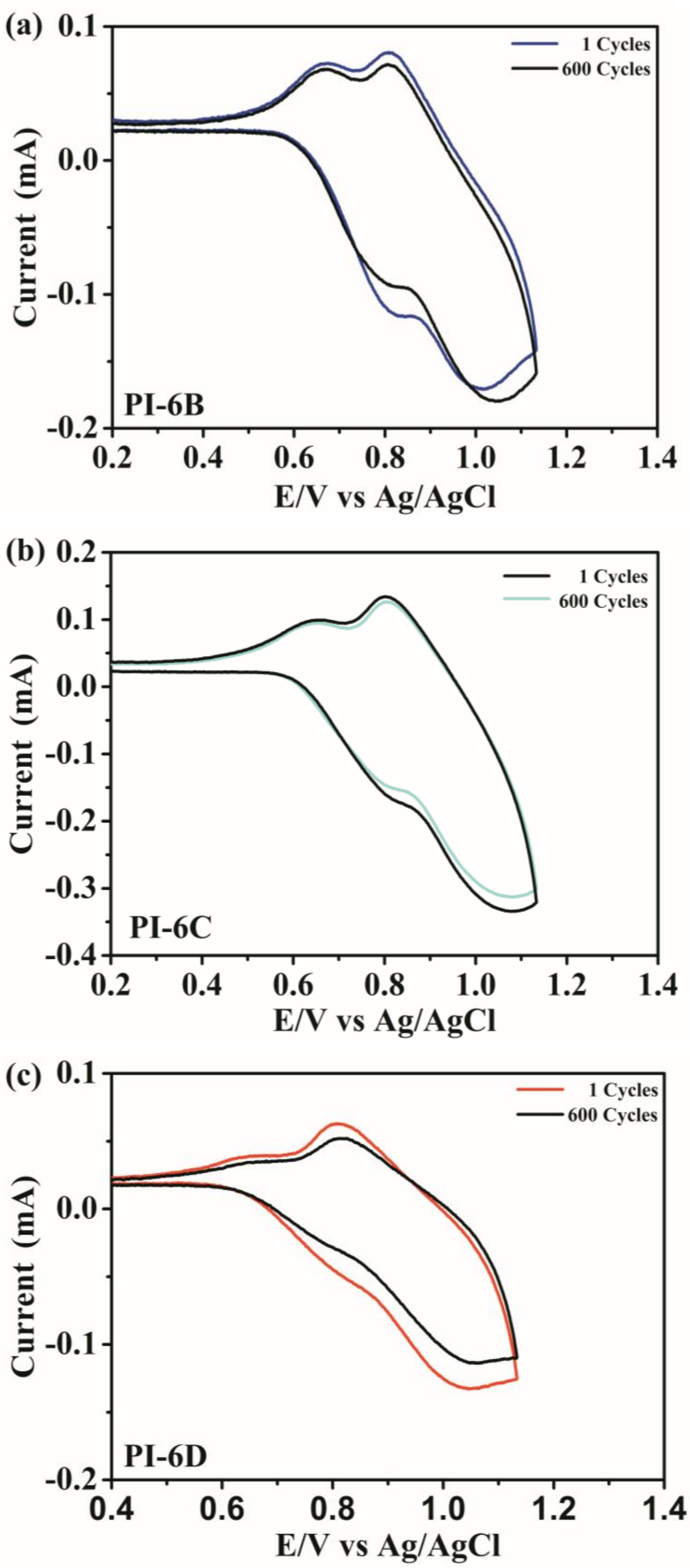
(a)
1 Cycles
600 Cycles
PI-6B
Current (mA)
E/V vs Ag/AgCl
0.1
0.0
-0.1
-0.2
0.2
0.4
0.6
0.8
1.0
1.2
1.4
(b)
1 Cycles
600 Cycles
PI-6C
Current (mA)
E/V vs Ag/AgCl
0.2
0.1
0.0
-0.1
-0.2
-0.3
-0.4
0.2
0.4
0.6
0.8
1.0
1.2
1.4
(c)
1 Cycles
600 Cycles
PI-6D
Current (mA)
E/V vs Ag/AgCl
0.1
0.0
-0.1
-0.2
0.4
0.6
0.8
1.0
1.2
1.4

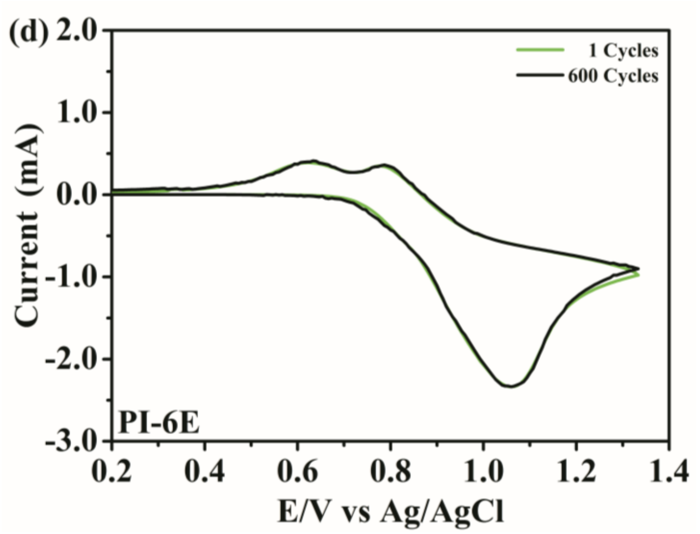

Fig. S3 Electrochemical stability of (a) **PI-6B,** (b) **PI-6C,** (c) **PI-6D** and (d) **PI-6E** films on an ITO-coated glass substrate in 0.1 M TBAP/CH_3CN solution at the scan rate of 50 mV·s^{-1} after 1000 switching by the CV method.

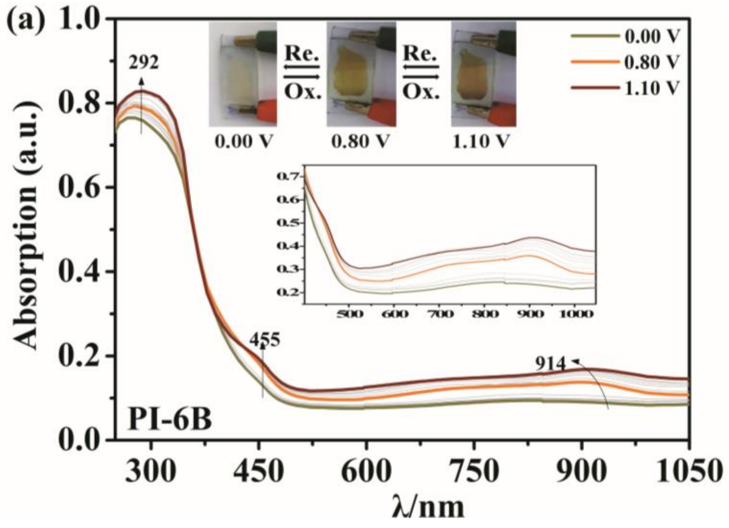

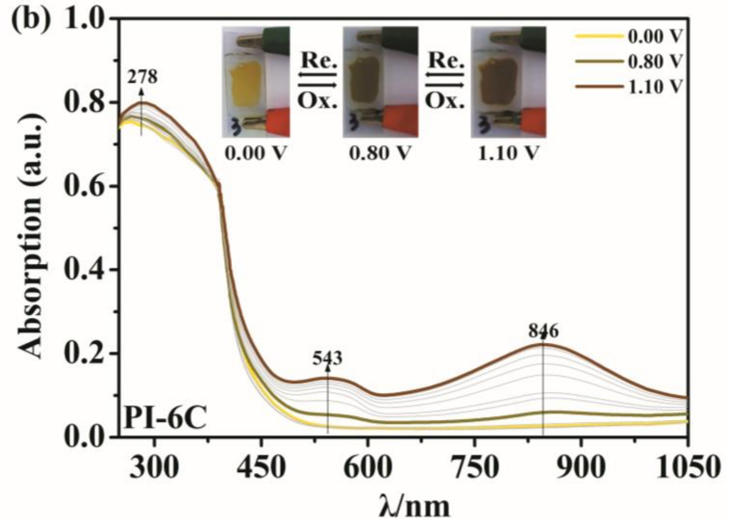

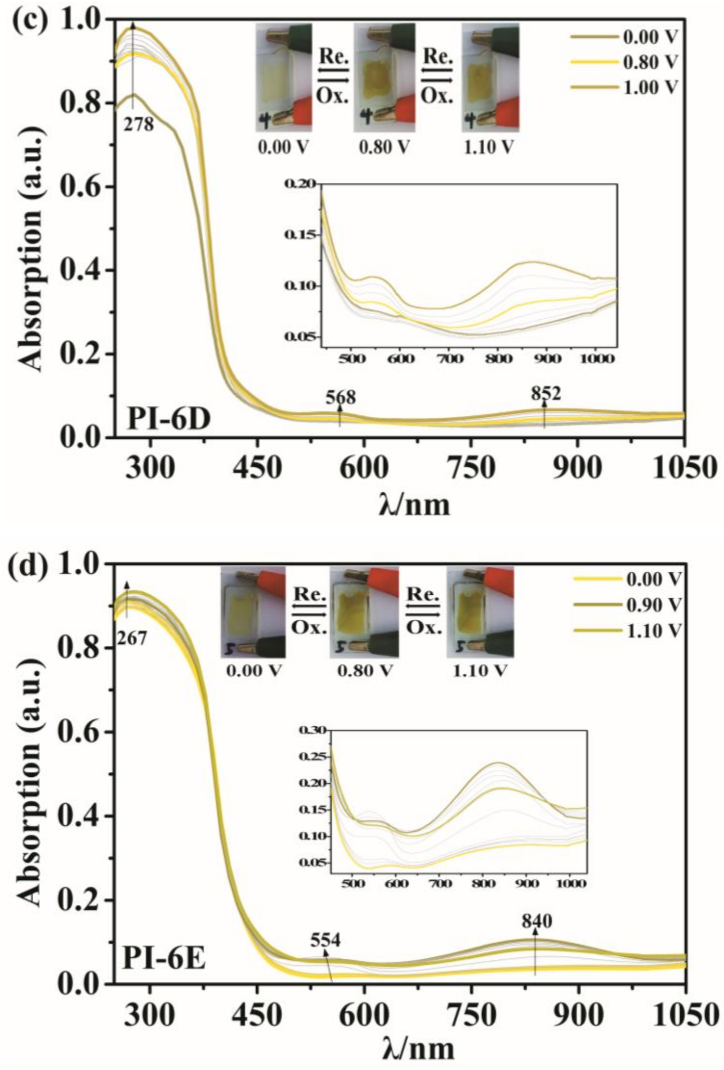

Fig. S4 Spectral change of (a) **PI-6B,** (b) **PI-6C,** (c) **PI-6D** and (d) **PI-6E** films on the ITO-coated glass substrate (0.1 M TBAP/CH_3CN solution as the supporting electrolyte) at different applied (V vs Ag/AgCl). The inset shows the photographic images of the film at indicated applied voltages.

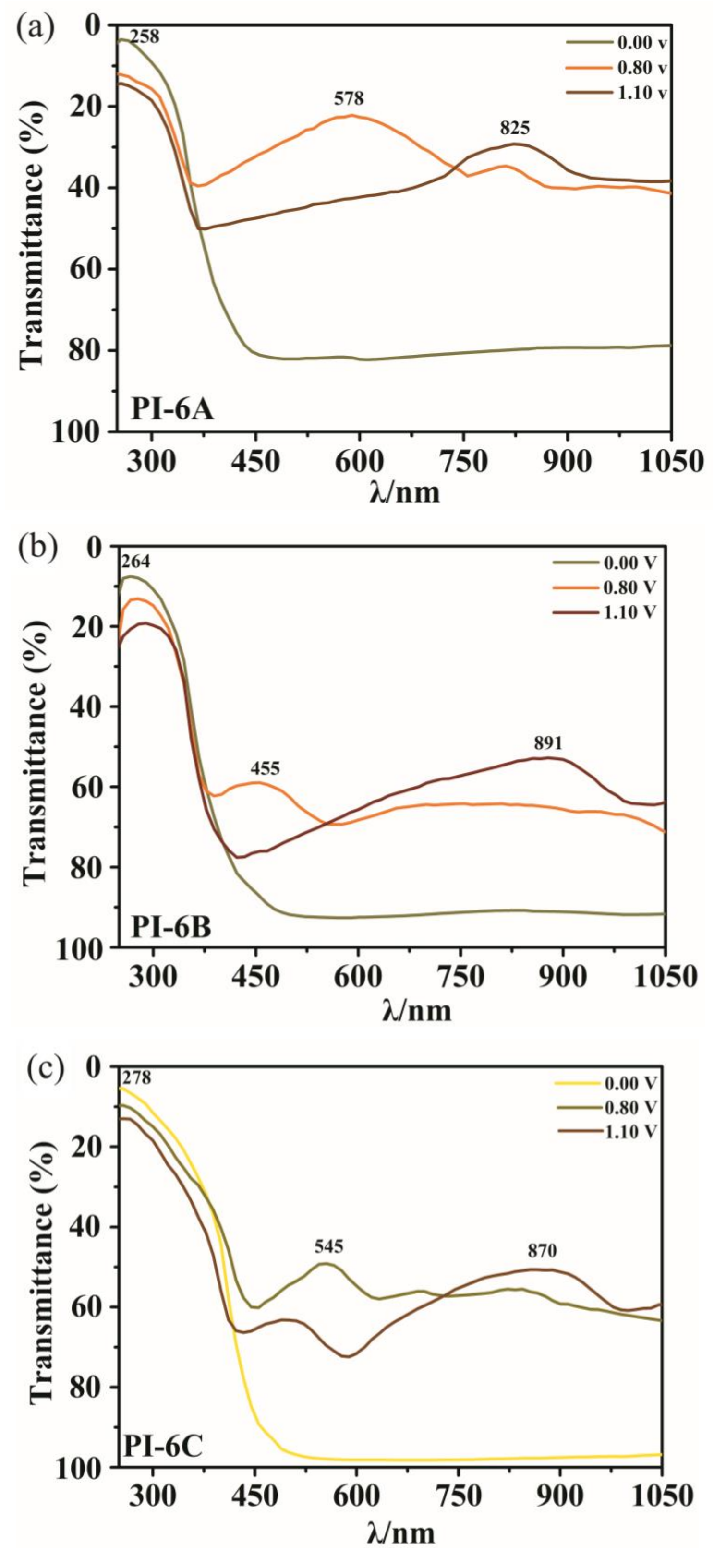
(a)
258
578
825
0.00 v
0.80 v
1.10 v
Transmittance (%)
PI-6A
λ/nm
(b)
264
455
891
0.00 V
0.80 V
1.10 V
Transmittance (%)
PI-6B
λ/nm
(c)
278
545
870
0.00 V
0.80 V
1.10 V
Transmittance (%)
PI-6C
λ/nm

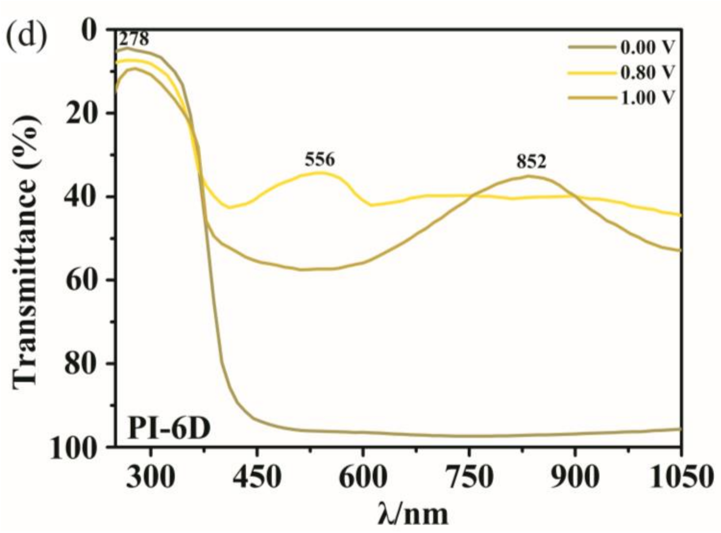

Fig. S5 EC behavior at different applied potentials (V vs Ag/AgCl) of **PI-6B, PI-6B, PI-6C, PI-6D** and **PI-6E** films (~210 nm in thickness) on the ITO-coated glass substrate (coated area: 1.0 cm× 4.0 cm) in 0.1 M TBAP/CH_3CN.

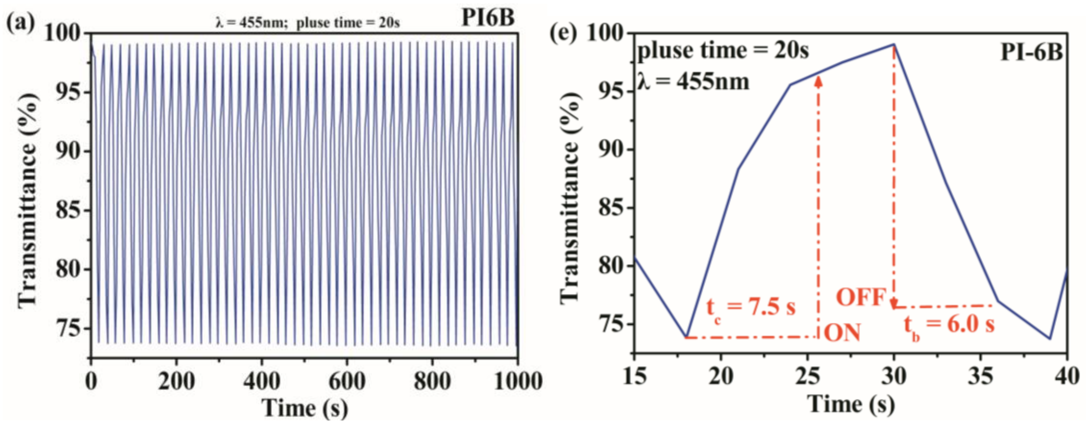

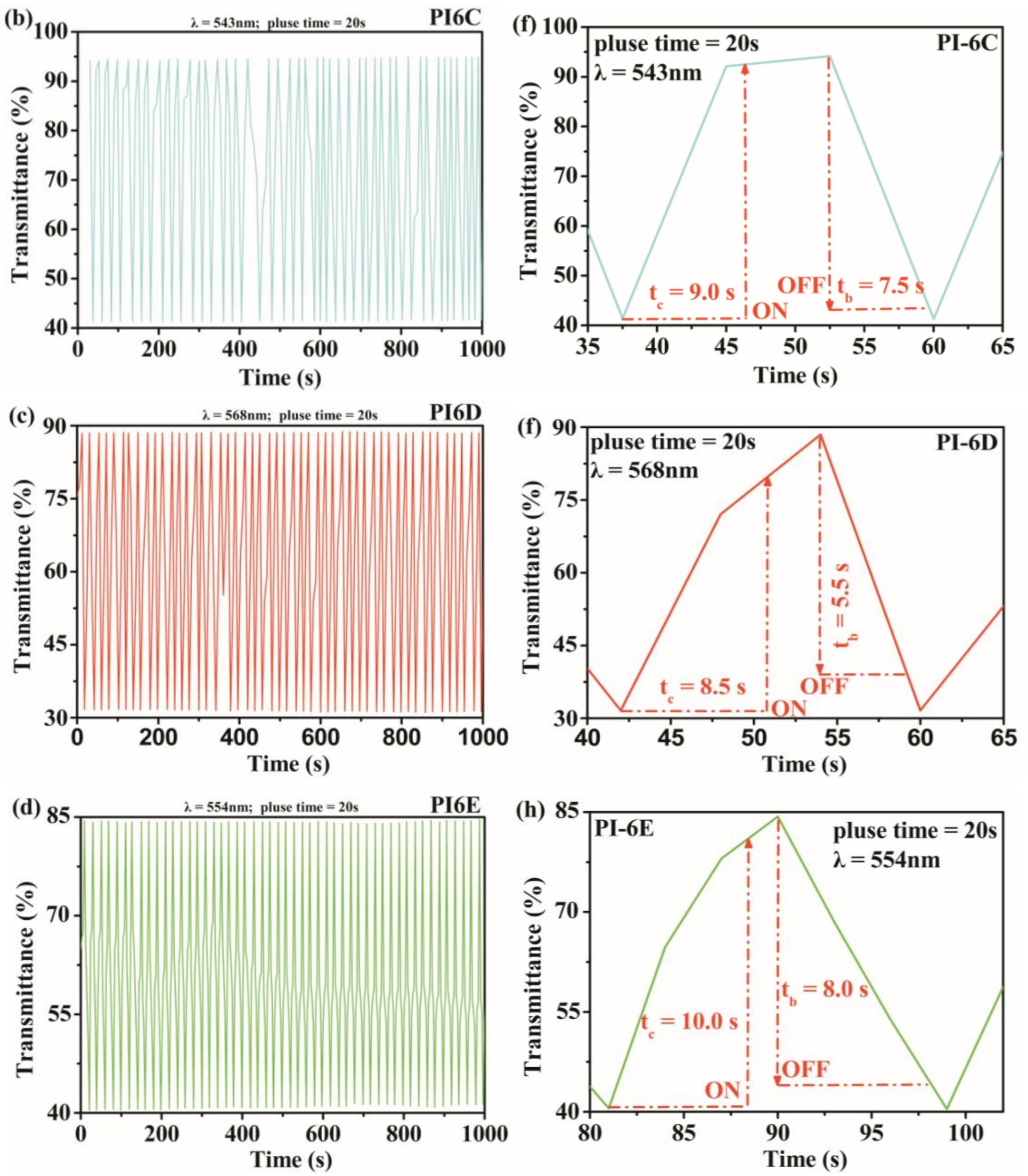

Fig. S6 Square-wave potential step absorptiometry and optical switching of **PI-6B - PI-6E** films.

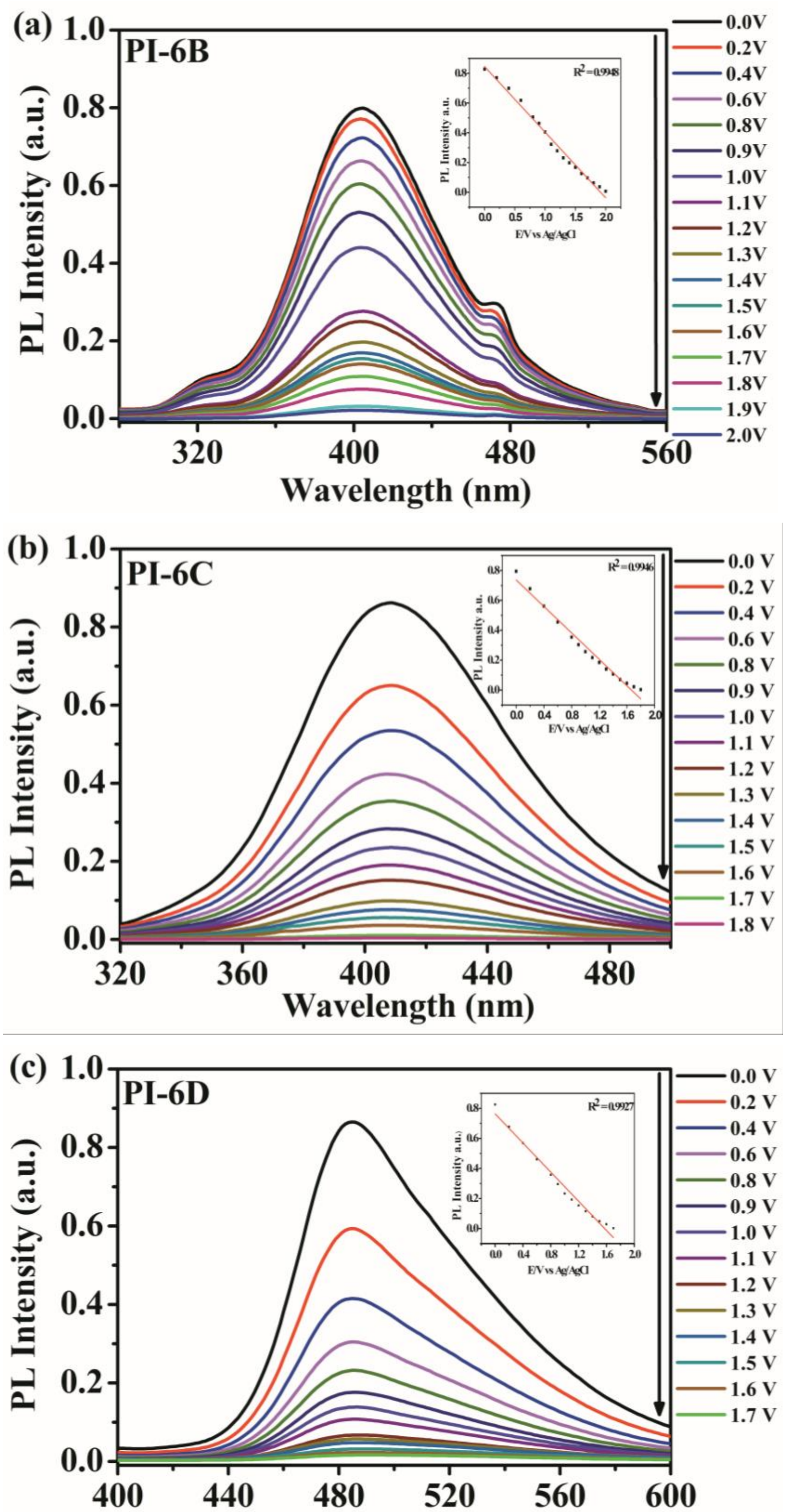
(a)
PI-6B
PL Intensity (a.u.)
Wavelength (nm)
0.0V
0.2V
0.4V
0.6V
0.8V
0.9V
1.0V
1.1V
1.2V
1.3V
1.4V
1.5V
1.6V
1.7V
1.8V
1.9V
2.0V
R2=0.9948
PL Intensity a.u.
E/V vs Ag/AgCl
(b)
PI-6C
PL Intensity (a.u.)
Wavelength (nm)
0.0 V
0.2 V
0.4 V
0.6 V
0.8 V
0.9 V
1.0 V
1.1 V
1.2 V
1.3 V
1.4 V
1.5 V
1.6 V
1.7 V
1.8 V
R2=0.9946
PL Intensity a.u.
E/V vs Ag/AgCl
(c)
PI-6D
PL Intensity (a.u.)
Wavelength (nm)
0.0 V
0.2 V
0.4 V
0.6 V
0.8 V
0.9 V
1.0 V
1.1 V
1.2 V
1.3 V
1.4 V
1.5 V
1.6 V
1.7 V
R2=0.9927
PL Intensity a.u.
E/V vs Ag/AgCl

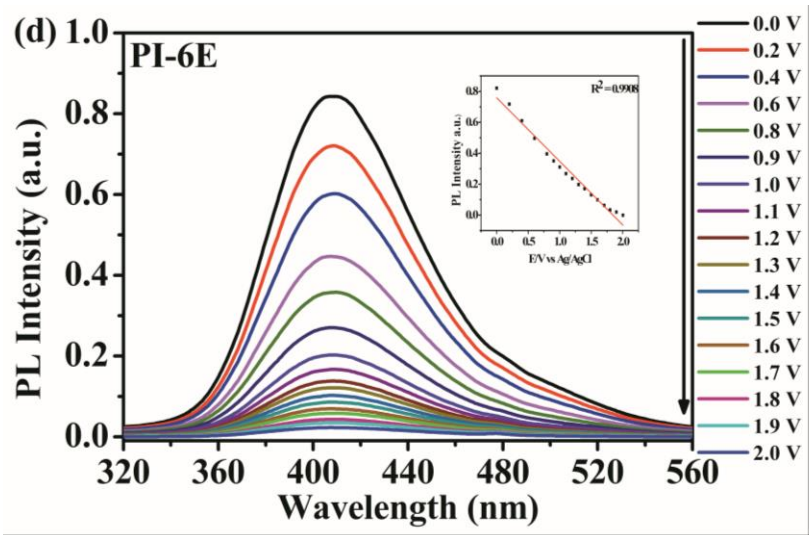

Fig. S7 PL spectra changes of (a) **PI-6B**, (b) **PI-6C**, (c) **PI-6D** and (d) **PI-6E** films upon different applied potentials.
